# Supplementary material for: Splice-Junction-Based Mapping of Alternative Isoforms in the Human Proteome
Source: Cell Rep. Author manuscript; Available in PMC 2020 Jan 15. (PMC6961840; doi:10.1016/j.celrep.2019.11.026)
Supplement: 2 [file NIHMS1546469-supplement-2.zip › DF1/PXD009737/TestisGluC/TestisGluC_19_MAP4_TSIFAGQNDPLKDSYVPLELAKE.pdf]

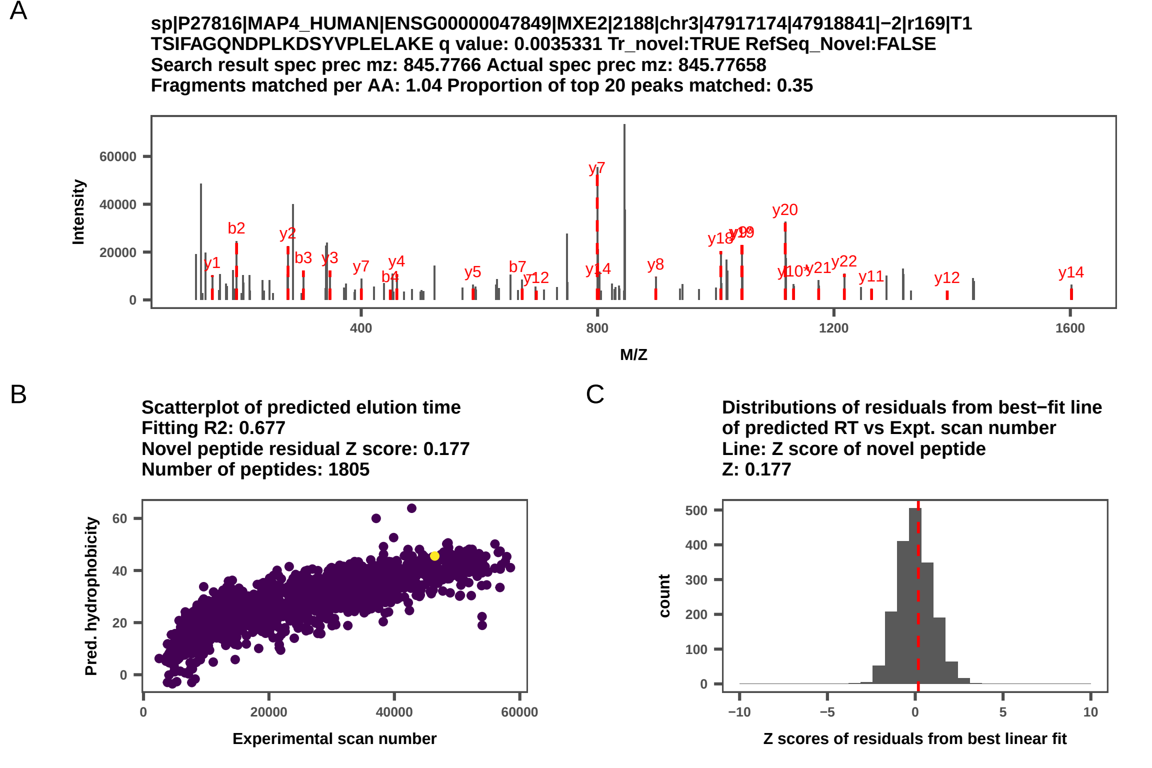
A
sp|P27816|MAP4_HUMAN|ENSG00000047849|MXE2|2188|chr3|47917174|47918841|−2|r169|T1
TSIFAGQNDPLKDSYVPLELAKE q value: 0.0035331 Tr_novel:TRUE RefSeq_Novel:FALSE
Search result spec prec mz: 845.7766 Actual spec prec mz: 845.77658
Fragments matched per AA: 1.04 Proportion of top 20 peaks matched: 0.35
Intensity
60000
40000
20000
0
y1
b2
y2
b3
y3
y7
y4
b4
y5
b7
y12
y7
y14
y8
y18
y20
y10
y21
y22
y11
y12
y14
400
800
1200
1600
M/Z
B
Scatterplot of predicted elution time
Fitting R2: 0.677
Novel peptide residual Z score: 0.177
Number of peptides: 1805
Pred. hydrophobicity
60
40
20
0
0
20000
40000
60000
Experimental scan number
C
Distributions of residuals from best−fit line
of predicted RT vs Expt. scan number
Line: Z score of novel peptide
Z: 0.177
count
500
400
300
200
100
0
−10
−5
0
5
10
Z scores of residuals from best linear fit
